# A New (Bloodless) Method of Removing Subserous Fibroids of the Uterus

**Published:** 1882-03

**Authors:** 


### A NEW (BLOODLESS) METHOD OF REMOVING SUBSEROUS FIBROIDS OF THE UTERUS.

At the last meeting of the gynecological section of the German Association of Naturalists and Physicians, (*Archiv f. Gynækologie* XVIII, 3,) Prof. Schroeder, of Berlin, read a paper on the management of the pedicle in Myotomy. His method is as follows:

The pedicle or neck of the tumor is firmly constricted by the elastic tube or band of an Esmarch's bandage, and the tumor cut off above it. There is a free discharge of blood from the engorged tumor, but presently the white, bloodless surface of the stump appears. This is then slightly excavated with the knife, so as to make a funnel-shaped depression in the stump. Care must be taken not to carry this excavation too deeply, as the elastic ligature might slip off. The edges of the stump are then approximated antero-posteriorly, and sutured with strong silk. The needles which must be very strong ones, are passed deeply through the parenchyma of the stump, avoiding the peritoneum. After approximating the edges, numerous smaller sutures are passed in the intervals between the larger ones, in order that there may be not the slightest gaping between the edges of the wound. After the edges have thus been brought accurately in contact, the peritoneum is drawn over the stump, and likewise stitched together with fine silk. The stump is thus placed entirely outside of the peritoneal cavity, and is only marked by the line of suture. The elastic ligature is then removed, and if any bleeding points remain, additional sutures are to be placed.

The operation done in this manner becomes entirely bloodless. The sutures must, however, be placed in close contiguity, and the approximation must be very exact. Owing to the absence of hemorrhage and the accessibility of the stump, the operation can be done with comparative rapidity. In one case where 56 sutures were applied, the operation was completed in an hour and a quarter. Schroeder has operated in nine consecutive cases by this method, with entire success. By this method, the tendency to abdominal hernias, which is very great where the clamp is used, is avoided.

